# Re-exposure to the hypobaric hypoxic brain injury of high altitude: plasma S100B levels and the possible effect of acclimatisation on blood–brain barrier dysfunction

**DOI:** 10.1007/s10072-016-2521-1

**Published:** 2016-02-29

**Authors:** C. D. Winter, T. Whyte, J. Cardinal, R. Kenny, E. Ballard

**Affiliations:** Kenneth Jamieson Department of Neurosurgery, Royal Brisbane and Women’s Hospital, Herston, QLD Australia; University of Queensland Centre for Clinical Research, Brisbane, QLD Australia; Department of Chemical Pathology, Royal Brisbane and Women’s Hospital, Herston, QLD Australia; School of Human Movement Studies, University of Queensland, St Lucia, QLD Australia; CAE Solutions, Iidabashi, Chiyoda-Kyu, Tokyo, Japan; Queensland Institute of Medical Research, Herston, QLD Australia

**Keywords:** S100B, Hypoxia, High altitude, Blood–brain barrier, Dexamethasone, Acclimatisation

## Abstract

Hypobaric hypoxic brain injury results in elevated peripheral S100B levels which may relate to blood–brain barrier (BBB) dysfunction. A period of acclimatisation or dexamethasone prevents altitude-related illnesses and this may involve attenuation of BBB compromise. We hypothesised that both treatments would diminish the S100B response (a measure of BBB dysfunction) on re-ascent to the hypobaric hypoxia of high altitude, in comparison to an identical ascent completed 48 h earlier by the same group. Twelve healthy volunteers, six of which were prescribed dexamethasone, ascended Mt Fuji (summit 3700 m) and serial plasma S100B levels measured. The S100B values reduced from a baseline 0.183 µg/l (95 % CI 0.083–0.283) to 0.145 µg/l (95 % CI 0.088–0.202) at high altitude for the dexamethasone group (*n* = 6) and from 0.147 µg/l (95 % CI 0.022–0.272) to 0.133 µg/l (95 % CI 0.085–0.182) for the non-treated group (*n* = 6) [not statistically significant (*p* = 0.43 and *p* = 0.82) for the treated and non-treated groups respectively]. [These results contrasted with the statistically significant increase during the first ascent, S100B increasing from 0.108 µg/l (95 % CI 0.092–0.125) to 0.216 µg/l (95 % CI 0.165–0.267) at high altitude]. In conclusion, an increase in plasma S100B was not observed in the second ascent and this may relate to the effect of acclimatisation (or hypoxic pre-conditioning) on the BBB. An exercise stimulated elevation of plasma S100B levels was also not observed during the second ascent. The small sample size and wide confidence intervals, however, precludes any statistically significant conclusions and a larger study would be required to confirm these findings.

## Introduction

S100B is a 21 kDa dimeric calcium-binding protein predominantly found in brain astrocytes [1] which regulates a variety of cellular functions [2]. There is an extensive body of literature regarding clinical conditions that have been associated with elevated S100B levels. A recent review [3] describes S100B associations with psychiatric disorders (schizophrenia, depression), sport (running, boxing, soccer), tumours (melanoma), multiple sclerosis, Alzheimer’s disease, cardiac disease, stroke and patients with traumatic brain injury or multi-trauma in the absence of brain injury. Peripheral S100B levels have also been noted to increase in patients with other hypoxic conditions such as obstructive sleep apnoea [4] and its postulated neuroprotective properties may also reflect increased expression secondary to post-injury reparative processes [5, 6]. Despite this many authors believe that peripheral S100B levels correlates with blood–brain barrier (BBB) dysfunction [7–9]. That CSF-serum albumin quotient (QA) is considered to be the gold standard for quantifying the extent of BBB dysfunction [10] and good correlation with serum S100B levels have been reported [9] provides further supportive data.

Although high-altitude related illnesses, such as acute mountain sickness (AMS), are thought to result from a multitude of processes, it is considered that blood–brain barrier (BBB) disruption is a significant contributor [11, 12]. Elevated peripheral S100B levels have been confirmed in volunteers at high altitude and this may reflect hypoxic opening of the BBB [13–15]. Furthermore, the vascular permeability factor vascular endothelial growth factor (VEGF) affects BBB function by altering tight junctions between endothelial cells and its increased expression in hypoxic conditions has been confirmed in animal studies [16] and mountaineers at high altitude [17]. The hypobaric hypoxia associated with altitude gain may induce VEGF release, a compromised BBB, cerebral vasogenic oedema and elevation of peripheral S100B. MRI findings of mild extracellular vasogenic oedema at simulated high altitude has also been described, suggestive of BBB compromise [18].

During the process of acclimatisation individuals exhibit specific cerebral physiological adaptive responses to reduced oxygen levels, such as increased cerebral blood flow (CBF) [19] and intracranial pressure [11]. Improved exercise tolerance, cognitive performance and symptoms of AMS following 16 days at high altitude have been reported [20]. Of more importance the latter group noted that after a period at low altitude the reduced incidence of AMS persisted upon re-ascent. Wu et al. [21] also reports improved AMS scores during re-exposure to 4500 m following long periods (up to 5 months) of low altitude, which correlated with improved oxygen saturations and lower resting HR. To our knowledge BBB dysfunction during re-exposure to high altitude has not been previously investigated. We hypothesised that the improvement in altitude related illnesses following acclimatisation may result from a beneficial effect on the BBB and this may correlate with diminished S100B levels. Our first paper [14] served to document two important stand-alone conclusions; that a statistically significant elevation in plasma S100B occurs with altitude but the S100B levels did not correlate with AMS. This companion paper from the same individuals at the same time points during the same ascent 48 h earlier allows us to compare two S100B data sets and comment on any possible effects of acclimatisation on S100B levels. When the participants ascended Mt Fuji 48 h earlier (i.e. the first ascent) they commenced at the same time of day, carried the same amount of weight (batteries, dry ice, medical equipment, etc.), took the same time interval to complete the distances between bleed-points, stayed overnight at the summit (exactly as per the second ascent) and descended at the same rate. We therefore felt that the two S100B data sets were comparable.

Dexamethasone is used for the prophylaxis and treatment of AMS and high altitude cerebral edema (HACE) [12] and this may involve attenuation of the BBB. The exact mechanism is unclear, though may involve changes in calcium-activated potassium channels [22] or reducing tight junction [23] or VEGF expression [24, 25]. We further hypothesised that volunteers prescribed dexamethasone would exhibit an additional reduction in S100B, in comparison to both the first ascent and with those who did not take dexamethasone in the second ascent. Studying the release of S100B in subjects at high altitude comprises a method of evaluating the effect of reversible hypoxic injury on the human BBB. Brain imaging (MRI) has been used in studies of simulated high altitude but clearly is not available to investigate the brain response at actual high altitude. Insights into the molecular responses by the brain to the hypobaric hypoxia of high altitude and re-exposure may potentially provide support for the development of novel therapeutic strategies for high attitude related illnesses and other hypoxic brain injuries.

As an addendum, a potential confounding factor for any such field study is the relationship between peripheral S100B levels and strenuous exercise. A recent systematic review [26] lists several studies confirming higher S100B levels post-exercise compared to pre-exercise values but also reports many studies which failed to confirm such a relationship. Ascending to 3700 m involves significant exertion and by comparing the two ascents we may also be able to comment on the possible phenomenon of exercise induced S100B elevation.

## Method

Following consent 12 healthy volunteers (gender ratio 1:1, age range 22–56) were recruited. The Lake Louise Questionnaire [27] and oxygen saturations (TuffSat, Datex Ohmeda) were completed at each bleed point. AMS was diagnosed when the participant experienced a headache and one other symptom to yield a score of 4 or more. On day 2 the group was driven to 1400 m, trekked to 2590 m (second blood test), and continued to the overnight accommodation at 3200 m. On day 3 we reached the summit (3700 m), remained for 2–3 h (blood test) and descended the mountain (blood test at 2590 m). Regular fluid intake was ensured in order to prevent dehydration. In summary, samples for S100B were taken at baseline (32 m above sea level), at 2590, at 3700 and then again at 2590 m during descent. The 12 subjects were randomly divided into a dexamethasone (*n* = 6) and a non-dexamethasone group (*n* = 6). The standard dose for the prevention or treatment of AMS was used (4 mg twice daily for 2½ days commencing at the beginning of the ascent).

At each bleed point 12 ml of venous blood (Li-Heparin tubes) was centrifuged for 3 min at 7200 rpm (4400*g*) using a portable centrifuge (StatSpin X3) (powered by 12 V batteries connected to an AC/DC inverter). The plasma was placed into dry ice (−80°), transported to Australia, stored at −80° and analyzed in duplicate with an automated chemiluminescence immunoassay (DiaSorin Liaison, Stillwater, MN, USA) with the Sangtec^®^ S100 analysis kit. For some of the participants the level at 2590 m was actually higher than the 3700 m level during ascent. The 2590 m value followed a fairly rapid ascent from 1400 to 2590 m (1190 m in 6–8 h) and it is well recognised that altitude sickness (and therefore potentially hypoxia induced BBB compromise and S100B level) relates to the rate of ascent as well as the final altitude gained. In addition, the 3700 m S100B level followed a period of rest at 3200 m after which we ascended to the summit and rested again for 2–3 h prior to the sample being taken. This summit S100B level may therefore have reflected a period of acclimatisation. Similarly, the 2590 m level during descent was taken within a few hours of the maximal period of hypobaric hypoxia on the summit. It was therefore deemed appropriate to use a mean of the three altitudes (2590 and 3700 on ascent, 2590 m on descent) for all subjects. The plasma S100B level was summarised by calculating a mean with 95 % confidence intervals at baseline and at high altitude.

Student’s *t* tests for independent or matched samples were used to compare groups or time points, respectively. The study has been reviewed and approved by the Royal Brisbane and Women’s Hospital Human Ethics Research Committee and carried out according to the National Statement on Ethical Conduct in Human Research (2007) produced by the National Health and Medical Research Council of Australia.

## Results

The results from this second ascent are presented and contrast with the parallel S100B data from the first ascent for the same 12 people [14]. The results for this current paper (the second ascent) note that 6 of the 12 participants were treated with dexamethasone (the ‘treated group’). The plasma S100B values reduced from 0.183 µg/l (95 % CI 0.083–0.283) at baseline to 0.145 µg/l (95 % CI 0.088–0.202) at altitude for the dexamethasone-treated group and from 0.147 µg/l (95 % CI 0.022–0.272) at baseline to 0.133 µg/l (95 % CI 0.085–0.182) at altitude for the non-treated group (Table 1). The reductions in S100B between baseline and altitude for this second ascent were not statistically significant for the treated (*p* = 0.43) or non-treated (*p* = 0.82) groups. There were no significant differences in plasma S100B levels at either baseline (*p* = 0.57) or at altitude (*p* = 0.70) between the treated or non-treated groups. Furthermore there was no statistically significant difference in the baseline plasma S100B level between the two ascents [all patients *p* = 0.077, treated *p* = 0.22, non-treated *p* = 0.27]. To summarise the first ascent S100B results, there was a significant (*p* < 0.001) increase in plasma S100B level from 0.108 µg/l (95 % CI 0.092–0.125) at baseline to 0.216 µg/l (95 % CI 0.165–0.267) at high altitude.

**Table 1:** Plasma S100B (µg/l) and oxygen saturations (%) during re-ascent of Mt Fuji (3700 m)

Parameter	Group	Baseline [mean (95 % CI)]	Average at altitude [mean (95 % CI)]	*p* value between altitudes
Plasma S100B (µg/l)	Treated (*n* = 6)	0.183 (0.083–0.283)	0.145 (0.088–0.202)	0.43
	Nontreated (*n* = 6)	0.147 (0.022–0.272)	0.133 (0.085–0.182)	0.82
*p* value between treatments		0.57	0.70	
Oxygen saturation (%)	Treated (*n* = 6)	97.3 (95.9–98.8)	92.2 (89.9–94.4)	0.001
	Nontreated (*n* = 6)	97.8 (97.4–98.3)	92.7 (90.4–94.9)	0.004
*p* value between treatments		0.41	0.70	

*Treated* dexamethasone treated group, *nontreated* non-dexamethasone group

During this ascent there was a statistically significant decrease in oxygen saturations from baseline to altitude for both the treated group [baseline 97.3 % (95 % CI 95.9–98.8), at altitude 92.2 % (95 % CI 89.9–94.4), *p* = 0.001] and for the non-treated group [baseline 97.8 % (95 % CI 97.4–98.3) at altitude 92.7 % (95 % CI 90.4–94.9), *p* = 0.004]. There were no significant differences at either baseline (*p* = 0.41) or at altitude (*p* = 0.70) in oxygen saturations between the treated and non-treated groups. There was no statistically significant difference in the baseline oxygen saturation levels between the two ascents (all participants *p* = 0.62, treated *p* = 0.46, untreated *p* = 0.36).

For completeness we compared the incidence of and S100B levels for AMS and non-AMS subjects. In the first ascent seven subjects experienced acute mountain sickness. During the second ascent the incidence of AMS reduced, affecting only three subjects (one from the dexamethasone group and two from the non-treated group), indicating that the process of acclimatisation had occurred. No correlation between plasma S100B levels and acute mountain sickness was evident during the first ascent and the same lack of correlation between S100B and AMS was found for the second ascent (Table 2). Given that there is no significant difference in plasma S100B levels between treatments and insufficient numbers of participants with AMS, comparisons were made between AMS and non-AMS sufferers only. The plasma S100B levels for the three AMS sufferers changed from 0.100 µg/l (95 % CI −0.029 to 0.229) at baseline to 0.127 µg/l (95 % CI −0.031 to 0.284) at high altitude which was marginally significant (*p* = 0.057) compared to the non-AMS sufferers who showed a non-significant (*p* = 0.35) change from 0.187 µg/l (95 % CI 0.102–0.272) at baseline to 0.143 µg/l (95 % CI 0.108–0.179) at altitude. There was no significant difference in plasma S100B levels between AMS and non-AMS groups at baseline (*p* = 0.23) or at altitude (*p* = 0.63). There was no significant difference in oxygen saturations between AMS and non-AMS participants at baseline (*p* = 0.88) or at altitude (*p* = 0.49). There was no significant difference between oxygen saturation levels at baseline between ascents for the non-AMS sufferers (*p* = 0.45) and AMS sufferers (*p* = 81).

**Table 2:** Plasma S100B and oxygen saturations (%) during re-ascent of Mt Fuji (3700 m); comparison of AMS and non-AMS subjects

Parameter	Group	Baseline [mean (95 % CI)]	Average at altitude [mean (95 % CI)]	*p* value between altitudes
Plasma S100B (µg/l)	AMS (*n* = 3)	0.100 (0.029–0.229)	0.127 (−0.031 to 0.284)	0.057
	Non-AMS (*n* = 9)	0.187 (0.102–0.272)	0.143 (0.108–0.179)	0.35
*p* value between treatments		0.23	0.63	
Oxygen saturation (%)	AMS (*n* = 3)	97.7 (96.2–99.1)	91.7 (86.5–96.8)	0.059
	Non-AMS (*n* = 9)	97.6 (96.7–98.4)	92.7 (91.0–94.3)	<0.001
*p* value between treatments		0.88	0.49	

*Treated* dexamethasone treated group, *nontreated* non-dexamethasone group, *AMS* acute mountain sickness

## Discussion

An elevation of peripheral S100B from baseline following the hypobaric hypoxia of high altitude has been previously documented and is thought to represent hypobaric hypoxic induced opening of the blood–brain barrier [13, 15]. The statistically significant increase in plasma S100B during ascent to the same altitude using the same group of volunteers [14] 48 h earlier allows us to comment on the potential effects of acclimatisation on the BBB when we compare those S100B levels with this second ascent. Although an elevation in S100B was not observed in this (second) ascent during re-exposure to the hypobaric hypoxia of high altitude for either the non-treated (acclimatised) or the dexamethasone treated (acclimatised and dexamethasone) groups, the hypothesis that S100B would reduce following acclimatisation could not be statistically confirmed in our study given the wide confidence intervals and small sample size. A larger study would be required to confirm these findings.

It would be easier to explain a lack of hypoxia-induced BBB compromise in our subjects during re-exposure to hypoxia if higher oxygen saturations were present compared to the first ascent. The reported improved oxygen saturations during re-exposure to hypobaric hypoxia [21, 28, 29] did not occur for our participants. The oxygen saturations at high altitude for the dexamethasone and the non-dexamethasone group were 92.2 and 92.7 % respectively, compared to 91.6 % for the first ascent, i.e. not significantly different (all patients *p* = 0.075, treated *p* = 0.36, untreated *p* = 0.14). Hypothesising on the mechanism(s) that may be involved in the improved BBB dysfunction following re-exposure to high altitude, extraneous to improved oxygen saturation, is speculative but may involve cerebral autoregulation, increased chemosensitivity or hypoxic preconditioning. Cerebral autoregulation (CA), the process by which CBF is maintained despite fluctuations in cerebral perfusion pressure (CPP), is impaired at high altitude and Subudhi et al. [20] recently confirmed persistence of dysfunctional CA on re-ascent. A failure of autoregulation may result in passive cerebral vasodilatation, a compromised BBB and increased vasogenic oedema. The degree of altered autoregulation may have been less severe 48 h later in our participants resulting in an improved BBB relative to the first ascent. A concept of enhanced hypoxic chemosensitivity has been suggested which posits that intermittent periods of hypoxia results in an augmented ventilatory response on re-exposure [30, 31]. A modified ventilation response to hypoxia or CO_2_ levels may theoretically adjust the balance of hypocapneic vasoconstriction versus hypoxic vasodilatation, local pH and bicarbonate concentrations. These dynamic processes all of which could potentially alter arteriolar and capillary vessel calibre and hydrostatic pressure changes with subsequent effects on the BBB during hypoxic re-exposure. The activation of specific genes is an important addition to adaptation to hypoxic conditions [31] and it may be that downstream gene products or transcription factors such as hypoxia-inducible-factor-1 (HIF-1) were still at play 48 h after being switched on and that an undiscovered mechanism attenuated the hypoxic induced BBB dysfunction. S100B has known neuro-protective properties, reducing glutamate excitotoxic damage [32] and enhancing neurogenesis [33] and the increased expression of S100B as part of a neuro-protective response to hypoxia may have been modified by acclimatisation.

Hypoxic pre-conditioning refers to the phenomenon of exposure to a moderate hypoxia/ischaemic (HI) insult providing increased resistance to a subsequent episode of HI [34, 35]. The first ascent may have acted as hypoxic pre-conditioning and this may have affected the BBB response to the second hypobaric hypoxic insult. Wick et al. [36] report an in vitro model enabling investigation into the possible molecular mechanisms involved in hypoxic preconditioning. Cerebellar granule neurons (CGN) preconditioned by sub-lethal exposure to reduced oxygen tension became resistant to successive injuries. The authors report elevated levels of VEGF and that the activation of VEGF/VEGF-receptor-2 (VEGFR-2), coupled with Akt/protein kinase B (PKB) phosphorylation and the subsequent enhancement of anti-apoptotic proteins may be integral to the neuroprotection afforded by hypoxic preconditioning. The enhanced expression of VEGF may also directly affect BBB permeability by causing loss of tight junction (TJ) proteins (such as occludin and claudin-5) and subsequent endothelial barrier disruption [37]. Following hypoxic injury to the brain the onset of a neuro-inflammatory cascade develops leading to increased release of cytokines, chemokines, reactive oxygen species and matrix metalloproteinases, all of which may cause disruption of the BBB. Significant protection secondary to hypoxic preconditioning in an in vivo model of neonatal HI injury has been described [38]. The authors report that the underlying mechanism may involve suppression of pro-inflammatory gene expression and a reduction in glial cell activation and the subsequent inflammation following re-exposure to hypoxia. Furthermore, Tang et al. [39] used a mouse model to examine the specific genomic response to ischemia following hypoxic preconditioning and compared this to gene expression following ischemia alone. They report the up-regulation of 27 genes mediating diverse function as sodium homeostasis, aquaporin-4 water channel expression, stress response and pro-inflammatory genes, all of which could conceivably affect blood–brain barrier function in the preconditioned ischemic animals. Many of these processes may account for the lack of a pronounced S100B response (potentially reflecting improved BBB function) during the second hypobaric hypoxic stimulus of the second ascent.

Our results also allow comment on the potential effects of dexamethasone on the BBB. A recent meta-analysis has confirmed the benefit of dexamethasone in the treatment and prevention of high altitude illnesses [40]. We hypothesised that dexamethasone would attenuate the hypobaric hypoxia induced BBB ‘leakiness’ and this would manifest as a lower S100B level in the dexamethasone treated group. However, there was no difference in high altitude plasma S100B level between the dexamethasone treated (*n* = 6) and non-treated groups (*n* = 6), we therefore could not confirm an additive effect of the steroid on attenuating the BBB compromise. An alternative possibility is that the process of acclimatisation overshadowed any potential effect of dexamethasone on S100B levels.

For any field study such as this we have to consider the effect of strenuous exertion on peripheral biomarker levels. Koh et al. [26] provides a review with some studies correlating elevated S100B levels with exercise while others did not. During our ascent the strenuous physical exertion was not associated with an increased S100B discounting any association. Comparing the S100B levels from the first ascent to this study we can conclude that if the elevated plasma S100B level in the first ascent was related to physical exertion, then a similar rise would be expected during the second ascent, but this was not observed. Our subjects were instructed to drink ad libitum reducing any potential dehydration effects. For completeness we analysed the S100B data for AMS and non-AMS subjects in the second ascent (Table 2). These results were in agreement with the first ascent, i.e. no correlation between AMS and S100B levels.

This study is open to the criticisms of any field study. Studies recruiting additional participants, measuring other biomarkers of BBB (e.g. matrix metalloproteinases) and the use of a hypoxic chamber with equivalent exercise are warranted. The effect of dexamethasone (without acclimatisation) may have been more instructive if we had repeated the ascent in 6 months, but we were limited by volunteer availability and funding restrictions. With hindsight it may have been prudent to combine the results from both ascents but the increase in S100B with altitude and the lack of correlation with AMS were important stand-alone conclusions. Additionally our S100B results relate to an ascent to 3700 m, and a more severe hypoxic stimulus (higher altitude) may have facilitated a statistical difference in the S100B levels between the dexamethasone treated and non-treated groups. Despite the limitations, to our knowledge the investigation of plasma S100B and by extension BBB function during re-exposure to high altitude, has not been previously explored. Analysing the molecular response of the BBB to hypobaric hypoxia and re-exposure to high altitude may elucidate secondary mechanisms that are common to many forms of brain injury such as stroke or subarachnoid haemorrhage.

## Conclusion

Our data suggests that the increase in plasma S100B during the first ascent was not observed in the second ascent for either the dexamethasone treated or non-treated groups. An exercise stimulated elevation of plasma S100B levels was also not observed during the second ascent. The small sample size and wide confidence intervals precludes any statistically significant conclusions and a larger study would be required to confirm these findings.
